# Reducing day 3 baseline monitoring bloodwork and ultrasound for patients undergoing timed intercourse and intrauterine insemination treatment cycles

**DOI:** 10.1186/s40738-021-00102-w

**Published:** 2021-04-30

**Authors:** Victoria O’Driscoll, Ilinca Georgescu, Irene Koo, Rebecca Arthur, Rita Chuang, Jillian Ann Dempsey, Giulia De Franco, Claire Ann Jones

**Affiliations:** 1 University of Toronto, Undergraduate Medical Education, 1 King’s College Circle, Room 3157, Toronto, ON M5S 1A8 Canada; 2 University of Toronto, Institute of Health Policy, Management, and Evaluation, 155 College Street, 4th floor, Toronto, ON M5T 3M6 Canada; 3 Schulich School of Medicine & Dentistry, Western University, 1151 Richmond Street, London, ON N6A 5C1 Canada; 4 Department of Obstetrics and Gynaecology, University of Toronto, 123 Edward Street, Suite 1200, Toronto, ON M5G 1E2 Canada; 5 Mount Sinai Fertility, Sinai Health System, 250 Dundas Street West, Suite 700, Toronto, ON M5T 2Z5 Canada

**Keywords:** Quality improvement, Resource stewardship, Timed intercourse, Intrauterine insemination, Infertility

## Abstract

**Background:**

In the current context of a global pandemic it is imperative for fertility clinics to consider the necessity of individual tests and eliminate those that have limited utility and may impose unnecessary risk of exposure. The purpose of this study was to implement and evaluate a multi-modal quality improvement (QI) strategy to promote resource stewardship by reducing routine day 3 (d3) bloodwork and transvaginal ultrasound (TVUS) for patients undergoing intrauterine insemination (IUI) and timed intercourse (IC) treatment cycles.

**Methods:**

After literature review, clinic stakeholders at an academic fertility centre met to discuss d3 testing utility and factors contributing to d3 bloodwork/TVUS in IC/IUI treatment cycles. Consensus was reached that it was unnecessary in patients taking oral/no medications. The primary intervention changed the default setting on the electronic order set to exclude d3 testing for IC/IUI cycles with oral/no medications. Exceptions required active test selection. Protocols were updated and education sessions were held. The main outcome measure was the proportion of cycles receiving d3 bloodwork/TVUS during the 8-week post-intervention period compared with the 8-week pre-intervention period. Balancing measures included provider satisfaction, pregnancy rates, and incidence of cycle cancellation.

**Results:**

A significant reduction in the proportion of cycles receiving d3 TVUS (57.2% vs 20.8%, *p* < 0.001) and ≥ 1 blood test (58.6% vs 22.8%, p < 0.001) was observed post-intervention. There was no significant difference in cycle cancellation or pregnancy rates pre- and post-intervention (*p* = 0.86). Treatment with medications, cyst history, prescribing physician, and treatment centre were associated with receiving d3 bloodwork/TVUS. 74% of providers were satisfied with the intervention.

**Conclusion:**

A significant reduction in IC/IUI treatment cycles that received d3 bloodwork/TVUS was achieved without measured negative treatment impacts. During a pandemic, eliminating routine d3 bloodwork/TVUS represents a safe way to reduce monitoring appointments and exposure.

**Supplementary Information:**

The online version contains supplementary material available at 10.1186/s40738-021-00102-w.

## Capsule

Implementation and evaluation of a multi-modal quality improvement strategy that effectively promoted resource stewardship by reducing routine day 3 bloodwork and transvaginal ultrasound at an academic fertility clinic.

## Introduction

Given the tight fiscal constraints on the Canadian healthcare system, it is crucial to identify ways of reducing unnecessary health care costs to reserve appropriate resources for high value health care [1]. In 2017, Choosing Wisely Canada reported that up to 30% of healthcare spending can be unnecessary, representing over 1 million unnecessary treatments and tests each year [2]. An analysis conducted in the United Kingdom (UK) has suggested that there is a lack of evidence for many of the interventions offered to couples experiencing infertility [3]. Fertility clinics are known to overuse testing, including the cyclical measurement of baseline cycle day 3 (d3) hormone bloodwork and transvaginal ultrasound (TVUS). Despite being common among fertility clinics, no essential role for d3 testing in patients undergoing intercourse (IC) or intrauterine insemination (IUI) treatments has been elucidated, and previous indications for excessive monitoring in the fertility setting, such as ovarian cysts and missed pregnancies, are now being questioned.

There is no literature available that supports the necessity of performing cycle monitoring on day 3. The current guideline provided by the National Institute for Health and Care Excellence in the UK recommends ultrasound monitoring during at least the first treatment cycle to identify the appropriate medication dose to minimize the risk of multiple pregnancy [4]. However, the guideline does not stipulate the value of d3 testing at which point there would be no information about the risk of multiples. Furthermore, it was originally thought to be important to assess for the presence of baseline ovarian cysts early in the cycle, however, studies have shown that baseline ovarian cysts do not actually impact pregnancy rate in women undergoing ovulation induction treatment [5]. While the majority of gynecologic outpatient/community clinics in the UK rarely or never follow the guideline to monitor a cycle with ultrasound, Canadian fertility clinics are frequent users of testing because of the ease of immediate access to on site sonography [6].

Unnecessary tests take time, include discomfort from venipuncture and TVUS, and may create unnecessary anxiety and stress from multiple early morning appointments. Additionally, unnecessary testing may lead to delays in treatment cycle starts, which can limit a patient’s opportunities to try to conceive. In the current context of a global pandemic when health care resources need to be allocated toward more urgent medical care and fertility clinics need to consider how to maintain physical distancing during treatment, it is imperative to consider the necessity of individual tests and eliminate those that have limited utility and may impose unnecessary risk of exposure.

The purpose of this study was to implement and evaluate a multi-modal quality improvement (QI) strategy to promote resource stewardship at an academic fertility clinic by reducing routine d3 bloodwork and TVUS for patients undergoing IUI and IC treatment cycles using oral medications or natural cycle, and identify root causes driving d3 testing. The primary outcome was the proportion of cycles that received d3 bloodwork and TVUS over the 8 weeks following the intervention compared with the 8 weeks prior to the intervention. Balancing measures included provider satisfaction and treatment outcomes cycle cancellation and pregnancy rate.

## Methods

This QI intervention involved multiple iterations of Plan-Do-Study-Act cycles over 8 weeks starting in April, 2019. Prior to the intervention, routine cycle monitoring bloodwork and TVUS took place on cycle day 3, 12, and depending on follicular growth, day 14–16. Day 3 baseline bloodwork and TVUS were identified as unnecessarily routine after an extensive literature review. Multi-disciplinary meetings were held to discuss a change from routine d3 bloodwork and ultrasound for women undergoing IC or IUI treatment cycles using a natural cycle or oral medications for ovarian stimulation. Key stakeholders, including physicians, nurse practitioner, quality manager, and clinic nurses were involved in these discussions. Fish-bone diagrams were created to identify perceived root causes (and potential predictors) of d3 testing overuse, as well as potential barriers to reducing d3 bloodwork and TVUS. Subsequent meetings with stakeholders were used to discuss implementation and adoption of the intervention.

The primary intervention consisted of a default electronic order set change. On April 26th, 2019, the default electronic order set used for patients undergoing IC, Donor-Intrauterine insemination (DI-IUI), and IUI using natural cycle or oral medications was changed from “YES” to “NO” d3 baseline bloodwork and TVUS. This was accompanied by a formal change in protocol that specified no routine d3 bloodwork or ultrasound for these patients. Exceptions were permitted but required active selection of baseline testing by the prescribing physician. Leading up to April 26th and onwards, regular emails were sent to clinic staff as a reminder of the protocol change. On July 31, 2019, a dialogue-based educational rounds was held with all clinic staff to discuss concepts of resource stewardship and the QI initiative.

Patients were included in the study if they underwent IC, DI-IUI, or IUI treatment cycles using a natural cycle or oral medication treatment protocol (Letrozole or Clomid) within the study dates March 1, 2019 to June 20, 2019. After stakeholder discussion, the decision was made to exclude patients receiving injectable gonadotropins from this intervention due to the financial cost of gonadotropins. Stakeholders wished to assess the efficacy of the intervention in the no/oral medications group prior to implementing the change for patients receiving the more expensive injectable medications. Treatment cycles were classified as either “pre” or “post” intervention depending on if the treatment cycle started before or after April 26th. For patients who underwent multiple treatment cycles within the 16-week time period, each treatment cycle was considered an individual data point in the analysis.

Data was collected from four centers associated with Mount Sinai Fertility, Toronto, Ontario, Canada by chart review of the clinic’s electronic medical record (EMR) system “eIVF”. The Data Analytics query function of eIVF was used to identify the total numbers of IC and DI-IUI/IUI treatment cycles that took place during the period of interest. Baseline data was retrospectively collected from the eight-week period immediately pre-intervention, and post-intervention data was collected prospectively for eight weeks immediately post change implementation by chart review. Prior to commencement, research ethics approval was granted by the Mount Sinai Hospital Research Ethics Board (REB#19–0107-E). Patients were informed about the change in protocol at the start of their cycle in the post-intervention period. Informed consent from patients was not required by the Research Ethics Board as this was a low-risk quality improvement intervention. There was no additional or reduced cost to patients participating in the intervention as all bloodwork and ultrasound monitoring was covered under public health care for IC and IUI cycles.

Data collected for each treatment cycle included: d3 bloodwork (beta-human chorionic gonadotropin (BhCG), progesterone, estradiol, FSH (follicle-stimulating hormone, luteinizing hormone (LH)) and TVUS, presence or absence of cyst on d3 TVUS, and d3 cycle cancellation and reason for cancellation. Data on potential root causes for d3 testing was collected: history of documented cysts, history of cancelled cycles, patient age, treatment protocol (IC or IUI/DI-IUI), treating physician, treatment centre, and type of medication. Protocol adherence was measured.

Descriptive statistics were presented for categorical and continuous variables. Chi-square or Fisher’s exact test was used to compare categorical variables. Generalized estimating equation (GEE) was applied to evaluate the association between hypothesized predictors of d3 testing and receiving d3 testing with adjustment for potential confounders. The proportion of treatment cycles that received d3 monitoring was compared pre- and post-intervention. Costs calculations were performed using Ontario Health Insurance Plan (OHIP) billing codes [7, 8]. Cost reductions after the intervention (represented by the column “Change” in Supplement 2/3) were calculated as follows: *OHIP billing code value for specific test ($) X (# of tests pre-intervention – # of tests post intervention) = Change*.

A convenience sample of physicians, nurse practitioners and nurses at Mount Sinai Fertility were invited to complete an anonymous survey regarding their experience with the QI intervention (Supplement 1). Survey data was presented using descriptive statistics.

## Results

A total of 1193 treatment cycles, representing data from 593 unique patients, met inclusion criteria for the study. Of these, 591 cycles took place in the pre-intervention period and 602 cycles took place in the post-intervention period. Significantly more IC cycles and fewer IUI cycles took place in the pre-intervention period compared with the post-intervention period (Table 1). A significantly higher proportion of patients with a past history of ovarian cyst and/or cancelled cycles were identified in the post-intervention period compared to the pre-intervention period (Table 1). There were no significant differences in stimulation protocol (*p* = 0.39) or mean age (*p* = 0.23) between intervention periods (Table 1).

**Table 1:** Descriptive statistics expressed as number (%) of treatment cycles pre- and post-intervention with comparisons made using Chi Square tests^a^ and Fischer Exact tests^b^

Total Treatment Cycles	Pre-Intervention (***n*** = 591)	Post Intervention (***n*** = 602)	***P*** value
Age (Standard Deviation)	34.68 (4.38)	34.98 (4.08)	0.23
History of Cyst	34 (5.8%)	94 (15.6%)	**< 0.01**^a^
History of Cancelled Cycle	47 (8.0%)	103 (17.1%)	**< 0.01**^a^
D3 BhCG	297 (*49.4%)*	107 (*18.1%)*	**< 0.001**^a^
D3 Estradiol and LH	350 (*58.2%)*	132 *(22.3%)*	**< 0.001**^a^
D3 Progesterone	331 *(55.1%)*	128 *(21.6%)*	**< 0.001**^a^
D3 FSH	281 *(46.8%)*	102 *(17.2%)*	**< 0.001**^a^
D3 TVUS	344 *(57.2%)*	123 *(20.8%)*	**< 0.001**^a^
**Timed Intercourse Cycles Only**	**314 (53.1%)**	**243 (40.4%)**	
**Stimulation Protocol**			0.43^b^
*- Natural Cycle*	13 (2.2%)	8 (1.3%)	
*- COS Clomid*	1 (0.2%)	2 (0.3%)	
*- COS Letrozole*	240 (40.6%)	197 (3.3%)	
*- Ovulation Induction Letrozole*	60 (10.2%)	36 (6.0%)	
D3 BhCG	168 (52.8%)	47 (19.7%)	**< 0.001**^a^
D3 Estradiol and LH	195 (61.3%)	62 (25.9%)	**< 0.001**^a^
D3 Progesterone	182 (57.2%)	60 (25.1%)	**< 0.001**^a^
D3 FSH	159 (50.0%)	51 (21.3%)	**< 0.001**^a^
D3 TVUS	190 (59.7%)	59 (24.7%)	**< 0.001**^a^
**IUI/D-IUI Cycles Only**	**277 (46.9%)**	**359 (59.6%)**	
**Stimulation Protocol**			
*- Natural Cycle*	34 (5.8%)	39 (6.5%)	0.58^a^
*- COS Letrozole*	203 (34.3%)	276 (45.8%)	
*- Ovulation Induction Letrozole*	40 (6.8%)	44 (7.3%)	
D3 BhCG	129 (45.6%)	60 (17.0%)	**< 0.001**^a^
D3 Estradiol	155 (54.8%)	70 (19.8%)	**< 0.001**^a^
D3 LH	155 (54.8%)	70 (19.8%)	**< 0.001**^a^
D3 Progesterone	149 (52.7%)	68 (19.3%)	**< 0.001**^a^
D3 FSH	122 (43.1%)	51 (14.4%)	**< 0.001**^a^
D3 TVUS	154 (54.4%)	64 (18.1%)	**< 0.001**^a^

**Fig. 1:**
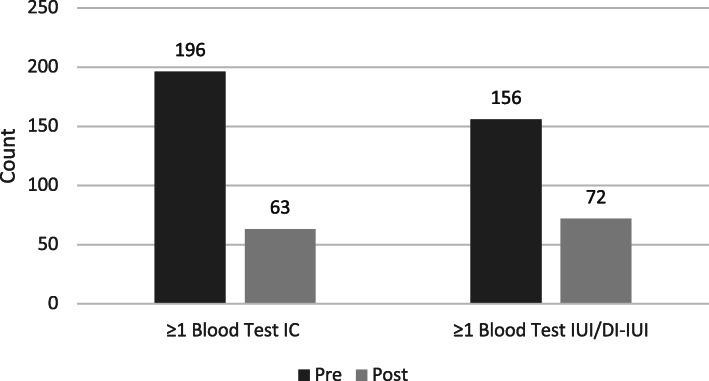
Number of IC and IUI/DI-IUI treatment cycles that received ≥1 d3 blood test (representing venipuncture) pre versus post intervention. This represents that the patient was brought in on day 3 for a bloodwork testing appointment. Significant reductions were observed post-intervention in cycles that received ≥1 blood test in both IC and IUI/DI-IUI treatment cycles (p < 0.001 and < 0.001, respectively)

A significant reduction in the proportion of cycles receiving d3 bloodwork and TVUS was observed post-intervention in both IC treatment cycles and IUI/DI-IUI treatment cycles, with each test experiencing a reduction of at least 28% for all tests (Table 1). The number of cycles that received ≥1 d3 blood test was also was significantly reduced (*p* < 0.001) (Fig. 1).

There was no difference in the rate of positive pregnancy tests identified on d3 or the rate of cycle cancellation between groups (Table 2). Although significantly fewer d3 TVUSs were performed in the post-intervention group, there was a significant increase in the percentage of ovarian cysts evident on the actively prescribed d3 ultrasounds in the post intervention group compared to the routine d3 ultrasounds pre-intervention (39 out of 130 vs. 62 out of 340 TVUS, *p* < 0.01). This likely represents a bias towards actively ordering d3 TVUS in women at higher risk for ovarian cysts (Table 2), resulting in a higher proportion of detected cysts in the post-intervention group. Reasons for cycle cancellation were similar between groups with the most common reason being patient-driven withdrawal (Table 2). There was no significant difference in the documented cycle outcomes; pregnant (intrauterine clinical gestation), not pregnant, or cancelled when comparing pre- and post-intervention groups, with some results still pending at the time of analysis (*p* = 0.86) (Table 2). Factors associated with an increased likelihood of receiving d3 bloodwork and/or TVUS were stimulation protocol, a history of ovarian cysts, a history of cancelled cycles, clinic site and one physician (Table 3).

**Table 2:** Balancing measures and clinical outcomes pre- and post-intervention calculated by Fischer exact test^a^ and Chi-square test^b^

Balancing Measure	Pre-Intervention Proportion (%)	Post-Intervention Proportion (%)	***P***-value
Beta-HCG positive on d3	5/294 (1.7%)	2/107 (1.9%)	1.00 ^a^
Ovarian cyst on d3 TVUS	62/340 (18.2%)	39/130 (30.0%)	**0.008** ^b^
Cycle Cancelled	87/591 (14.7%)	85/602 (14.1%)	0.68^b^
• Cycle Cancelled on d3	8/87 (9.2%)	5/85 (5.9%)	0.44^b^
Cycle outcome			0.86 ^b^
• Cancelled	87/591 (14.7)	85/602 (14.1)	
• Not pregnant	420/591 (71.2)	428/602 (71.1)	
• Pregnant/clinical intrauterine gestation	43/591 (7.3)	41/602 (6.8)	
• None recorded	40/591 (6.8)	48/602 (8)	
Reasons for Cycle Cancellation			0.1^b^
• Patient withdrawal	47.1	39.3	
• Premature or missed ovulation	14.1	33.3	
• Low ovarian response	4.7	3.6	
• High ovarian response or risk of multiples	4.7	4.8	
• Ovarian cyst	5.9	3.6	
• Other	23.5	15.5

**Table 3:** Relative risk and corresponding 95% CI for having d3 blood test and ultrasound by factor

	Bloodwork	TVUS
Intervention (Post vs. Pre)	**0.37** [0.32,0.44]**	**0.35** [0.30,0.41]**
Age		
< 35	1.00 [1.00,1.00]	1.00 [1.00,1.00]
36–39	1.02 [0.88,1.17]	1.02 [0.88,1.17]
> 39	0.97 [0.80,1.19]	0.92 [0.76,1.13]
Cycle Type		
IC	1.00 [1.00,1.00]	1.00 [1.00,1.00]
IUI	0.88 [0.77,1.01]	0.89 [0.77,1.02]
DI-IUI	0.88 [0.57,1.35]	0.92 [0.60,1.41]
Protocol		
Natural	1.00 [1.00,1.00]	1.00 [1.00,1.00]
COS (clomid + Letrozole)	**2.86** [1.66,4.92]**	**4.55** [2.65,7.84]**
Ovulation Induction (Letrozole)	**3.48** [2.03,5.97]**	**5.36** [3.12,9.18]**
Hx cysts		
No	1.00 [1.00,1.00]	1.00 [1.00,1.00]
Yes	**1.37** [1.11,1.70]**	**1.45** [1.17,1.80]**
Hx cancelled		
No	1.00 [1.00,1.00]	1.00 [1.00,1.00]
Yes	**1.23** [1.00,1.53]*	1.17 [0.95,1.46]
Doctor		1.00 [1.00,1.00]
1	1.00 [1.00,1.00]	
2	0.82 [0.58,1.14]	0.79 [0.57,1.11]
3	1.21 [0.93,1.58]	1.21 [0.93,1.57]
4	1.23 [0.97,1.54]	1.19 [0.94,1.49]
5	1.08 [0.84,1.39]	1.14 [0.89,1.47]
6	0.95 [0.74,1.23]	1.02 [0.79,1.31]
7	**1.35** [1.01,1.81]*	**1.41** [1.05,1.88]*
8	0.90 [0.48,1.67]	0.88 [0.47,1.64]
Centre		
Downtown Toronto	1.00 [1.00,1.00]	1.00 [1.00,1.00]
Vaughan	**0.66** [0.50,0.86]**	**0.68** [0.52,0.89]**
North York	**0.76** [0.59,0.96]*	**0.74** [0.59,0.95]*
Mississauga	1.68 [0.94,3.00]	1.70 [0.95,3.03]
Observations	1144	1144
Pseudo *R*^2^	0.024	0.027

Exponentiated coefficients; 95% confidence intervals in brackets

* *p* < 0.05, ** *p* < 0.01

The cost of d3 TVUS and bloodwork for IC cycles was calculated to be $12,035.60 over the 8-week pre-intervention period compared to $3773.22 in the post-intervention period, representing a cost reduction of $8262.38 in the 8 weeks after the intervention (Supplement 2). For DI-IUI/IUI cycles, we estimated costs using the same OHIP calculation because of the difficulty in calculating true costs for individual blood tests and ultrasound through the Ontario Fertility Program where a flat fee is paid to clinics for all services related to an IUI cycle. We estimated costs for d3 TVUS and bloodwork for IUI cycles to be $9675.96 during the pre-intervention period and $3791.77 during the post-intervention period, representing an estimated cost reduction of $5884.19 (Supplement 3).

We received survey responses from 18 nurses, 4 physicians, and 1 nurse practitioner. Greater heterogeneity in responses was observed for the statement regarding provider attitude towards whether testing is performed in excess for patients undergoing intercourse and IUI cycles with or without oral medications. Sixty one percent of respondents agreed with this statement. Seventy-four percent of providers felt satisfied with the reduction in testing, and 70% agreed with the statement that the intervention had changed their practice (Supplement 4). There was wider heterogeneity in responses to questions about patient anxiety or patient satisfaction with the reduction in testing (Supplement 4).

## Discussion

This QI study demonstrated a successful reduction in d3 monitoring bloodwork and TVUS without an increase in measured negative effects to patients or clinicians. The components of this intervention included a change in the default EMR ordering system followed by a formal protocol change, and staff education. The intervention was successful in demonstrating a significant reduction in d3 BhCG, FSH, LH, estradiol, and progesterone tests, as well as TVUS, in the eight weeks post-intervention in both IC and IUI/DI-IUI groups. Each of the aforementioned tests were reduced by a minimum of 28% post-intervention. This resulted in an absolute OHIP costs savings of $8262.38 for IC cycles during the 8-week intervention period with additional estimated cost savings of $5884.19 for IUI cycles.

The EMR intervention was the most effective intervention in our study, compared with protocol change and educational interventions. A common barrier to the reduction of unnecessary testing is clinician behavior [9]. Clinicians tend to avoid deviating from traditional medical practices out of habit and fear of litigation or of missing an uncommon but important diagnosis, resulting in slow adoption of newer clinical guidelines. A reliance on automated order sets to support complex workflows, such as those found in fertility treatment centers, have reduced the level of conscious engagement on behalf of clinicians when ordering tests. Systems-level changes targeting EMRs have been shown to be some of the most effective single interventions used to modify test ordering behaviors by physicians [10, 11]. EMR changes such as limiting the number of tests listed, eliminating pre-selected orders, changing the default option, and forced functions, have all been shown to successfully reduce unnecessary laboratory testing and interventions [12–15]. Advantages to EMR modifications include inexpensive implementation, easy sustainability, and immediate effectiveness [16]. Changes to default settings, similar to what was done in this study, are a particularly attractive means of reducing unnecessary testing since they require active decision making on the part of physicians in order to ‘opt in’ to testing, and assigns a greater responsibility and stewardship awareness to those physicians who choose to deviate from the protocol [17]. Previous studies have also shown that default settings, with the option for provider override when clinically indicated, can reduce unnecessary testing without compromising patient care [18, 19].

In the post-intervention period, d3 testing was more likely to be ordered in those patients with an oral medication protocol, a history of cysts, treatment at the Mississauga site, and in study participants who were the patient of one particular physician. Specific reasons for reduced uptake of the intervention by this particular physician were not explored in this study, however clinician survey demonstrated that most providers accepted the protocol change. The finding that the Mississauga site assigned more tests is likely reflective of the fact that the physician who ordered significantly more d3 tests primarily practiced at this site. Day 3 bloodwork (though not TVUS) was also associated with a history of cancelled cycles. Missing a clinically unrecognized pregnancy prior to commencing fertility medications which are contraindicated in pregnancy was considered to be a significant barrier to changing to no d3 bloodwork/TVUS identified by physicians prior to commencing the study [20–24]. The overall prevalence of a positive serum BhCG on d3 was similar between groups, with less than 2% in the pre-intervention population (5 of 294), and less than 2% (2 of 107) when d3 BhCG was actively selected for in the post-intervention population (*p* = 1). While uncommon, we have sought to mitigate this risk by advising patients to take a home urine test each cycle prior to commencing any fertility medications.

Having a history of an ovarian cyst was associated with d3 testing. This was due to a perceived poorer response to medications in the presence of a cyst, concern that fertility medications may stimulate further growth of cysts, and that it may be difficult to differentiate a follicle from a cyst later on without baseline monitoring among physicians. However, previous studies have demonstrated no difference in pregnancy rates between women with baseline ovarian cysts compared to women without [5]. The incidence of ovarian cysts observed on pre-intervention d3 TVUS was 18.2% which is similar to what has been previously published, but it rose to 30% of d3 TVUS in post-intervention cycles, correlating with the increased incidence of d3 TVUS ordered in patients with a history of ovarian cysts [25]. Despite there being more d3 testing in patients with a history of ovarian cysts, this was an uncommon reason for cycle cancellation. Our study provides further evidence against using the presence of cysts to guide d3 testing as it does not affect treatment decision-making. Although not statistically significant, 33.3% of cancelled cycles in the post-intervention group were due to missed ovulation, compared to 14.1% in the pre-intervention group. For some of these patients, d3 TVUS may have allowed for sooner detection of early follicular recruitment, however, it is unlikely that the final outcome of cancelled treatment cycle would have changed with this information.

Based on the survey carried out to assess health care provider attitudes, most nurses and physicians had a positive attitude towards reducing d3 bloodwork/TVUS in patients undergoing IUI/IC cycles, and felt encouraged to change their practice. Additionally, most respondents did not perceive increased confusion on the part of our patients in response to the protocol change. When trying to gauge whether this intervention had an effect on patient anxiety or satisfaction, results were more heterogeneous, perhaps reflective of ambiguity in our respondents’ experience in assessing the patient experience. One limitation of our study is a lack of survey data on patient perspectives regarding the reduction in d3 testing.

Other limitations include the short time period for evaluation of the QI intervention, which prevented us from evaluating intervention sustainability. However, changes that are embedded directly into the EMR and that do not require additional effort by staff are likely to be sustained. Furthermore, treatment plans may have been created by physicians prior to the protocol change resulting in more d3 appointments during the post-intervention period than would occur over time. Finally, not all treatment cycle outcomes had been finalized in the EMR at the time of data collection. Future steps include measuring the sustainability of the intervention, and identifying other areas of redundancy in fertility testing and treatment and applying a similar multi-modal intervention.

Strengths of this study included a large number of treatment cycles examined, and a strong EMR intervention that is easy to implement and generalizable to other fertility clinics. Our study demonstrates that reducing d3 bloodwork and TVUS is safe for patients and can have significant impacts on cost-savings and resource management. We demonstrated a total cost reduction of $8262.35 in the 8 weeks post-intervention for IC cycles using the Ontario Schedule of Benefits Physician Services under the Health Insurance Act and the Schedule of Benefits for Laboratory Services [7]. IUI cycles were more difficult to measure as they are mostly covered under the Ontario Fertility Program under which $725 is paid to the fertility clinic to cover operating costs of the treatment cycle, so we were only able to estimate a savings of $5884.19 for IUI cycles post-intervention. Removing unnecessary testing also freed up time for physicians, sonographers and nurses to see other patients during that time, which cannot be easily quantified.

Most importantly, this simple intervention resulted in potential savings in cost, time and convenience for patients. The impact of avoiding unnecessary testing for patients should not be considered lightly. Patients undergoing fertility treatment frequently undergo numerous tests which can be stressful, time-consuming, and cause physical discomfort. Furthermore, previous studies have demonstrated increased psychological distress in patients with unnecessary interventions [26, 27]. Therefore, reducing unnecessary testing has the potential to have psychological benefits.

In a time of a global pandemic, where physical distancing is a public health requirement, fertility clinics must find avenues to safely reduce the number of patients coming into the clinic for tests/procedures while providing safe and quality care. Our study demonstrated that d3 bloodwork/TVUS can be safely and effectively removed from the monitoring of IC/IUI cycles in women taking oral medications or using a natural cycle. This easy intervention can help manage patient volumes during this crisis and has prompted us to evaluate what other tests can be eliminated while still maintaining safe and effective patient care.

## Conclusion

This study has demonstrated an efficacious multi-modal QI intervention to reduce d3 bloodwork and TVUS to promote resource stewardship in the field of fertility medicine. As healthcare providers, we are responsible for the provision of high-quality, high-value care –performing appropriate testing at the appropriate time. Reducing unnecessary d3 testing not only saves healthcare dollars, it saves time and reduces burden for both patients and providers while reducing risk of COVID-19 exposure.

## Supplementary Information

**Additional file 1: Supplement 1.** Clinician Survey. **Supplement 2.** Cost Calculations for day 3 Laboratory Tests and TVUS for IC Treatment Cycles. **Supplement 3.** Cost Calculations for Day 3 Laboratory Tests and TVUS for IUI/DI-IUI Treatment cycles. **Supplement 4.** Clinician Survey Results.

## Data Availability

The datasets used and/or analysed during the current study are available from the corresponding author on reasonable request.

## Abbreviations

BhCG: Beta-human chorionic gonadotropin
CI: Confidence Interval
D3: Day 3
DI-IUI: Donor Intrauterine Insemination
EMR: Electronic Medical Record
FSH: Follicle-stimulating Hormone
GEE: Generalized estimating eq.
IC: Timed Intercourse
IUI: Intrauterine Insemination
LH: Luteinizing Hormone
OHIP: Ontario Health Insurance Plan
QI: Quality Improvement
TVUS: Transvaginal Ultrasound
