# Supplementary material for: Reducing day 3 baseline monitoring bloodwork and ultrasound for patients undergoing timed intercourse and intrauterine insemination treatment cycles
Source: Fertil Res Pract. 2021 Apr 30;7:11. doi: 10.1186/s40738-021-00102-w (PMC8085474; doi:10.1186/s40738-021-00102-w)
Supplement: Supplementary file 1 — Additional file 1: Supplement 1. Clinician Survey. Supplement 2. Cost Calculations for day 3 Laboratory Tests and TVUS for IC Treatment Cycles. Supplement 3. Cost Calculations for Day 3 Laboratory Tests and TVUS for IUI/DI-IUI Treatment cycles. Supplement 4. Clinician Survey Results. [file 40738_2021_102_MOESM1_ESM.docx]

Reducing day 3 baseline monitoring bloodwork and ultrasound for patients undergoing timed intercourse and intrauterine insemination treatment cycles

Victoria O’Driscoll^1,2^, Ilinca Georgescu^3^, Irene Koo^4,5^, Rebecca Arthur^4,5^, Rita Chuang^4^, Jillian Ann Dempsey^5^, Giulia De Franco^5^, Claire Ann Jones^4,5^

1. University of Toronto, Undergraduate Medical Education.

2. University of Toronto, Institute of Health Policy, Management, and Evaluation

3. Schulich School of Medicine & Dentistry, Western University

4. University of Toronto, Department of Obstetrics and Gynaecology

5. Mount Sinai Fertility, Sinai Health System

**Additional Material**

**Supplement 1:** Clinician survey

Please state whether you agree or disagree with the following statements using the 10-point Likert scale below in which 1=strongly disagree, 5=neither agree nor disagree, and 10=strongly agree.

1. Patients undergoing intercourse and IUI cycles with oral medications or in natural cycles undergo too many blood and ultrasound tests at fertility clinics.

-------------------------------------------------------------------------------------------

1 2 3 4 5 6 7 8 9 10

1. I am satisfied with the reduction in blood and ultrasound tests for women undergoing intercourse and IUI cycles by the interventions introduced during this study period.

-------------------------------------------------------------------------------------------

1 2 3 4 5 6 7 8 9 10

1. Patients are satisfied with a reduction in the number of blood and ultrasound tests performed.

-------------------------------------------------------------------------------------------

1 2 3 4 5 6 7 8 9 10

1. The reduction in the number of blood tests and ultrasound has created more confusion and anxiety for me in communicating with patients.

-------------------------------------------------------------------------------------------

1 2 3 4 5 6 7 8 9 10

1. A reduction in the number of blood tests and ultrasounds has created more anxiety for patients.

-------------------------------------------------------------------------------------------

1 2 3 4 5 6 7 8 9 10

1. This study and the interventions from this study have changed my practice.

-------------------------------------------------------------------------------------------

1 2 3 4 5 6 7 8 9 10

**Supplement 2:** Cost calculations for day 3 laboratory tests and TVUS for IC treatment cycles

| Lab Test  (OHIP CODE) | Number of Tests  Pre-Intervention | Number of Tests  Post-Intervention | Cost per test  ($) | Cost Pre-Intervention ($) | Cost Post-Intervention ($) | Change ($) |
| --- | --- | --- | --- | --- | --- | --- |
| BhCG  (L318) | 168 | 47 | 3.29 | 552.72 | 154.63 | 398.09 |
| Estradiol (L310) | 195 | 62 | 7.08 | 1380.60 | 438.96 | 941.64 |
| FSH  (L315) | 159 | 51 | 4.61 | 732.99 | 235.11 | 497.88 |
| LH  (L328) | 195 | 62 | 4.71 | 918.45 | 292.02 | 626.43 |
| Progesterone  (L331) | 182 | 60 | 8.12 | 1477.84 | 487.20 | 990.64 |
| TVUS  (J164) | 190 | 59 | 36.70 | 6973.00 | 2165.30 | 4807.70 |
| **Total** |  |  |  | 12035.60 | 3773.22 | 8262.38 |

**Supplement 3:** Cost calculations for day 3 laboratory tests and TVUS for IUI/DI-IUI treatment cycles

| Lab Test | Number of Tests  Pre Intervention | Number of Tests  Post Intervention | Cost per test  ($) | Cost Pre-Intervention ($) | Cost Post-Intervention  ($) | Change  ($) |
| --- | --- | --- | --- | --- | --- | --- |
| BhCG  (L318) | 129 | 60 | 3.29 | 424.41 | 197.40 | 227.01 |
| Estradiol  (L310) | 155 | 70 | 7.08 | 1097.40 | 495.60 | 601.80 |
| FSH  (L315) | 122 | 51 | 4.61 | 562.42 | 235.11 | 327.31 |
| LH  (L328) | 155 | 70 | 4.71 | 730.05 | 329.70 | 400.35 |
| Progesterone  (L331) | 149 | 68 | 8.12 | 1209.88 | 522.16 | 657.72 |
| TVUS  (J164) | 154 | 54 | 36.70 | 5651.80 | 1981.80 | 3670.00 |
| Total |  |  |  | 9675.96 | 3791.77 | 5884.19 |

**Supplement 4:** Clinician survey results
